# Tutorial on High-Definition Map Generation for Automated Driving in Urban Environments

**DOI:** 10.3390/s22187056

**Published:** 2022-09-18

**Authors:** Jinseop Jeong, Jun Yong Yoon, Hwanhong Lee, Hatem Darweesh, Woosuk Sung

**Affiliations:** 1 School of Mechanical System and Automotive Engineering, Chosun University, Gwangju 61452, Korea; 2 Graduate School of Informatics, Nagoya University, Furo-cho, Chikusa-ku, Nagoya 464-8603, Japan

**Keywords:** autonomous driving, high-definition map, localization, path planning

## Abstract

High-definition (HD) mapping is a promising approach to realize highly automated driving (AD). Although HD maps can be applied to all levels of autonomy, their use is particularly beneficial for autonomy levels 4 or higher. HD maps enable AD systems to see beyond the field of view of conventional sensors, thereby providing accurate and detailed information regarding a driving environment. An HD map is typically separated into a pointcloud map for localization and a vector map for path planning. In this paper, we introduce two separate but successive HD map generation workflows. Of the several stages involved, the registration and mapping processes are essential for creating the pointcloud and vector maps, respectively. To facilitate the readers’ understanding, the processes of these two stages have been recorded and uploaded online. HD maps are typically generated using open-source software (OSS) tools. CloudCompare and ASSURE, as representative tools, are used in this study. The generated HD maps are validated with localization and path-planning modules in Autoware, which is also an OSS stack for AD systems. The generated HD maps enable environmental-monitoring vehicles to successfully operate at level 4 autonomy.

## 1. Introduction

A high-definition (HD) map is a geospatial map for automated navigation, which is different from traditional maps for human drivers (see Table 1).

**Figure 1:**
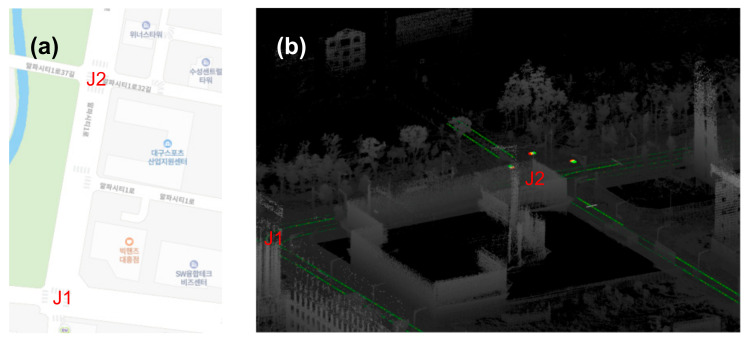
Shape comparison of (**a**) digital and (**b**) HD maps. J denotes a junction.

Digital maps have been widely used to assist drivers in navigation. However, for highly automated navigation, such as levels 4 or higher, digital maps are considered to be insufficient in terms of accuracy and comprehensiveness. Therefore, accurate and detailed HD maps have recently emerged (see Figure 1 and Table 2). In contrast to conventional digital maps, HD maps exhibit several unique features, such as three-dimensional (3D) lane-level representations to assist automated driving (AD) modules in localization, perception, and path planning. In doing so, an HD map is considered as not simply a navigation map but a sensor that can provide 360° non-line-of-sight awareness to support an AD system.

Multiple layers of geographical information are required for the AD system, in a form known as the local dynamic map (LDM) [3]. Although the relevant standards have certain differences, an LDM is generally divided into four layers according to the time intervals in which the dynamic information changes (see Table 3). In this configuration, an HD map corresponds to the bottommost layer.

**Figure 2:**
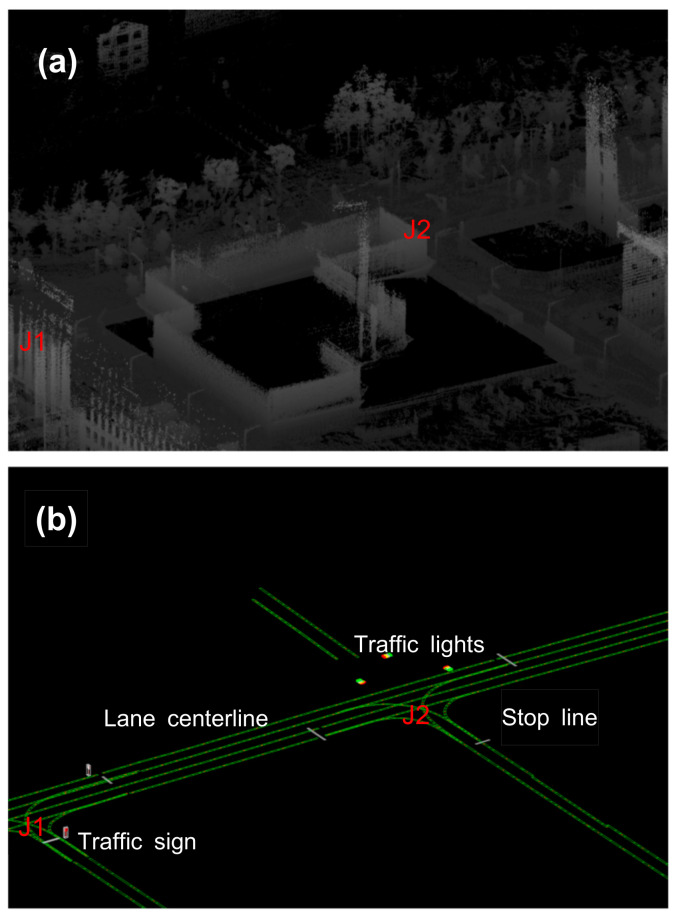
Shape comparison of (**a**) pointcloud and (**b**) vector data in the map. J denotes a junction.

In general, an HD map can be separated into pointcloud and vector maps. A vector map, also known as a road network map, represents the georeferenced position of the objects of interest in a driving environment (lanes, traffic signs and lights, etc.) with points, lines, and polygons, whereas a pointcloud map represents the 3D shape of objects with points (see Figure 2).

Different HD map formats, particularly for the physical storage format (PSF) of a vector map, have been developed to suit different requirements. Consider, for example, the Aisan vector map, which is well-known as the proprietary PSF used for Autoware.AI. Autoware.AI is a robot operating software (ROS)-based open-source software platform that provides a rich set of AD modules. Another variant of Autoware.AI, Autoware.Auto, has emerged as the next-generation successor. Autoware.Auto is based on ROS 2 to exhibit enhanced functional safety capabilities. For the sake of brevity, Autoware.AI, which was used in this study, is referred to as Autoware in the remaining paper.

Although the Aisan vector map was adopted successfully, the Autoware user group was encouraged to diversify the PSF to render Autoware more expandable. Therefore, several PSFs were comparatively evaluated (Table 4), and Lanelet2 was selected as a short-term alternative on the condition that OpenStreetMap (OSM) XML is used as the format for reading and writing map data. Additionally, OpenDRIVE was selected as the ultimate alternative, which is widely used in existing industry standards together with the Navigation Data Standard (NDS) Open Lane Model. Consequently, from version 1.13 of Autoware onwards, Lanelet2 support has been added to all ROS nodes that use the Aisan vector map. In later versions, Lanelet2 will be used as a default vector map option, and the Aisan vector map will be deprecated.

**Figure 3:**
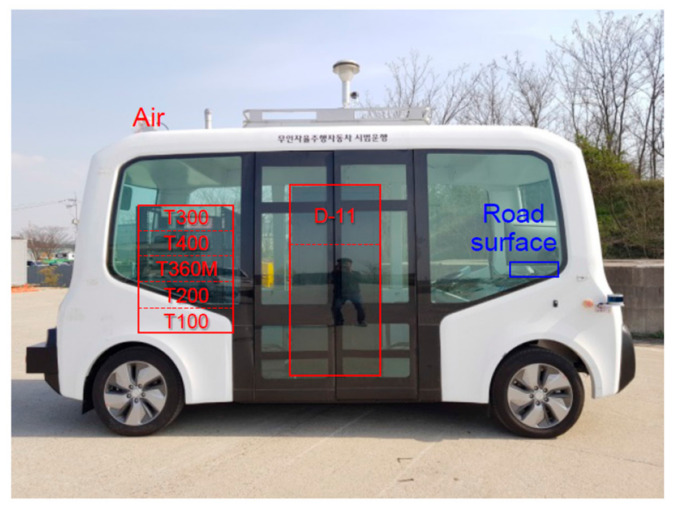
Developed vehicle and equipped environmental monitoring instruments.

This study was part of a regulation-free special zone project led by the Ministry of SMEs and Startups and Gwangju-si in Korea [6]. The zone refers to an area designated to allow companies to test their innovative technologies without regulatory restrictions. Gwangju-si has been designated as a zone for unmanned special-purpose vehicles. The operation of vehicles developed for different purposes, such as street cleansing, garbage collection, and environmental monitoring has been demonstrated in this zone. We helped develop the AD system of vehicles for environmental monitoring. The vehicle was deployed in the Pyeong-dong industrial complex in Gwangju-si with onboard instruments to monitor the quality of the environment (Figure 3 and Table 5).

Because the vehicle was required to operate unattended, the AD system was aimed at achieving level 4 autonomy. The key difference between levels 3 and 4 is that level 4 systems do not require human interaction in the operation unless a system failure occurs. Because no attendant was available to take control over the faulty system, a higher degree of reliability must be ensured for all modules of the AD system from perception to control. To this end, an HD map, as a set of pointcloud and vector maps, was used in this study. Owing to the limited operational area of the Pyeong-dong industrial complex, we could perform mapping in advance, inhering certain features from the prior maps. In general, pointcloud maps facilitate localization to decrease the positioning error to less than several centimeters without using any real-time kinematic (RTK)-enabled global navigation satellite system (GNSS) receiver. Once an ego-vehicle has been localized in the pointcloud map, the vector map can be used for path planning relative to the lane centerline further ahead than is possible with on-board multi-channel light detection and ranging sensors (LiDARs), thereby enhancing ride comfort and safety. The vector map also facilitates traffic light recognition (TLR) to enhance the recognition rate while decreasing the computational cost without vehicle-to-infrastructure (V2I) communication.

In Korea, domestic HD map standards have been established by the National Geographic Information Institute (NGII). The PSF of the vector map established by the NGII is unique in terms of the data model structure and elements (see Table 6).

Because the AD system we developed is based on Autoware, the NGII vector map format must be converted to that supported by the platform. Map format conversion is necessary to customize an open-source software platform. Considering the requirements of the platform side, i.e., OpenPlanner, HD map generation workflows were established in this study. OpenPlanner is the most widely used path-planning module within Autoware. With the emergence of version 2.0 of OpenPlanner, the module has become considerably more advanced in terms of its capabilities of supporting diverse HD map formats, introducing a human–machine interface, predicting the trajectories of other vehicles by estimating their intention, and generating the trajectories of an ego-vehicle via kinematics-based motion simulations [8]. The module integrates global, local, and behavior planners that jointly use the road network map to generate local trajectories based on a global path and manage discrete behaviors such as avoiding dynamic obstacles and stopping before a stop line or traffic lights. Among the various vector map formats that the module supports, we selected ASSURE KML, a PSF optimized for OpenPlanner and expected to be highly compatible with its map editor, ASSURE. A high compatibility is expected because the selected planners, map format, and map editor have been created and maintained by the same group at Nagoya university [9].

The remaining paper is organized as follows. We present two separate but successive workflows: Section 2 and Section 3 describe the workflows for the pointcloud and vector map generation, respectively.

## 2. Pointcloud Map

**Figure 4:**
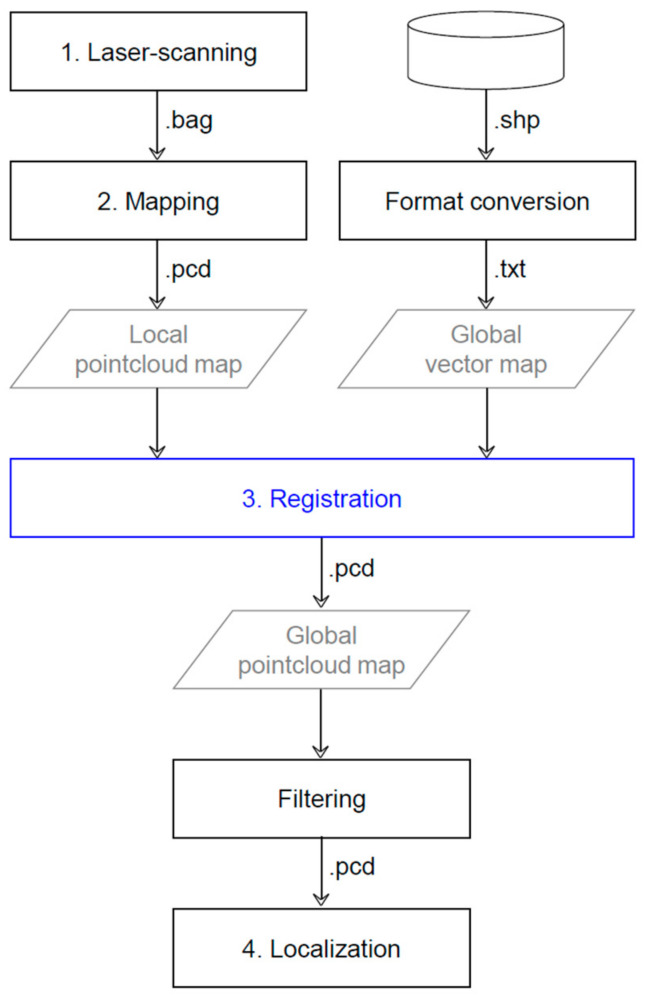
High-level flowchart outlining key stages in pointcloud map generation.

The workflow of the pointcloud map generation consists of four key stages: laser scanning, mapping, registration, and validation (see Figure 4 and Table 7).

### 2.1. Laser Scanning

The first stage is to record the scanned pointcloud data. Launch Autoware. Although it is not necessary to use Autoware, it is considerably easier to use Runtime Manager than entering commands directly in a terminal. In the Sensing tab, launch the driver node to publish the scanned pointcloud as/points_raw. Check off the box of Ouster OS1-64. Confirm that the scanned pointcloud is published with RViz. Click the RViz button. In the Displays panel on the left, change the Fixed Frame under Global Options to velodyne, and check that/points_raw is displayed. To modify the camera pose, change Type of the camera in the Views panel on the right. Record the scanned pointcloud by clicking the ROSBAG button. ROSBAG is a file format designed to log ROS messages. In the generated pop-up window, click the Refresh button to update the list of available ROS topics. Click the Ref button and type a ROSBAG filename. Scroll through the list of the updated ROS topics and check off the/points_raw box. Click the Start button to start ROSBAG recording.

**Figure 5:**
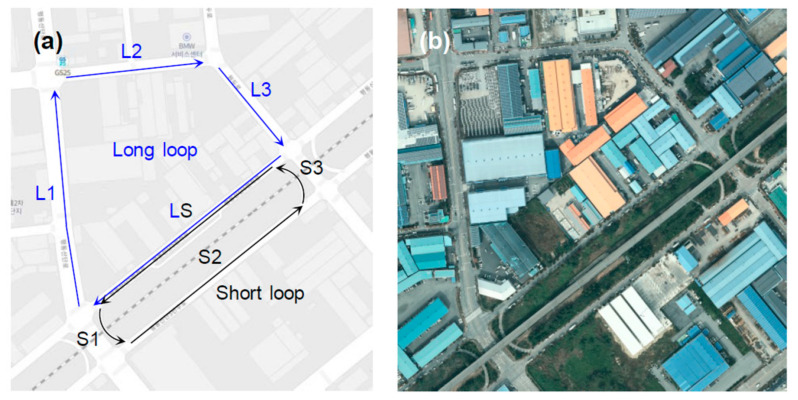
(**a**) Long and short loops in the operational area represented on (**b**) satellite map.

While recording the scanned pointcloud data, drive the vehicle smoothly and consistently around the operational area, preferably in a closed loop. The pose of the vehicle should not suddenly change within the loop. The rest of the mapping process is implemented under the assumption that the scanned pointcloud data are continuous because sudden changes may disrupt the operation of the normal distribution transform algorithm. Instead of a dedicated mobile mapping system, a LiDAR (Ouster OS1-64) fixed on the back of the roof of the vehicle is used. In this configuration, the LiDAR to be used for localization is also used for surveying. The operational area centers around two closed loops sharing a one-way road between them (see Figure 5 and Table 8), and the loops must be laser scanned individually. After driving through one loop, click the Stop button to finish the ROSBAG recording. The two separate pointclouds relative to a local frame are merged on a global frame in the third stage. Laser scanning must ideally be repeated in different scenarios, for example, in the opposite direction, at higher velocities and in an open loop, enabling the production of another set of scanned pointcloud data. The scanned pointcloud data, different than those used to generate the pointcloud map in the next stage, can be used to validate the generated pointcloud map in the final stage.

### 2.2. Mapping

The second stage is to align the recorded pointclouds with each other. Launch Autoware. The actual usage of Autoware for mapping has been screen recorded in [10]. In the Computing tab, launch the mapping node by checking off the approximate_ndt_mapping box. This node is a variant of the ndt_mapping node, and a comparison between the two mapping nodes is presented in Table 9. Play the recorded pointcloud data by clicking the Ref button in the Simulation tab and specifying the relevant ROSBAG file. Verify the information regarding the recorded topics, mostly/points_raw, and click the Play button. Confirm that the recorded pointcloud is published and correctly aligned.

**Figure 6:**
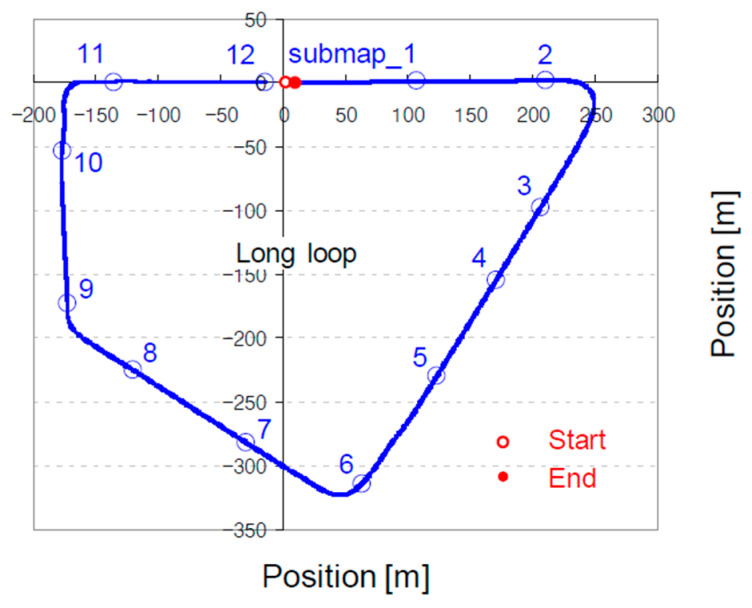
Progress of approximate_ndt_mapping inspected using the CSV file generated with PCD files.

If the mapping process is being appropriately implemented, the scanned pointclouds will be arranged in a sequential order, reproducing the path that the vehicle traversed in the previous stage. The path can be visualized with RViz. However, it is preferable to draw a chart with the generated CSV file (see Figure 6) because visualization with RViz may introduce a computational burden in the mapping process. The aligned pointcloud is saved as a pointcloud data (PCD) file. PCD is a file format designed to store 3D pointcloud data. Even after the playback has finished, the mapping process is typically in progress owing to its computationally intensive nature. Unlike the ndt_mapping node, the approximate_ndt_mapping node produces a PCD file every 2–3 min in the mapping process. The produced PCD files are named submap_*x*, according to their creation order. When a PCD file is no longer created, the mapping process can be considered to have ended. Notably, the completion of the process cannot ensure the quality of the generated pointcloud map. The chart must ideally be prepared to determine if the mapping process has been successfully completed (see Figure 6).

### 2.3. Registration (Remapping)

**Figure 7:**
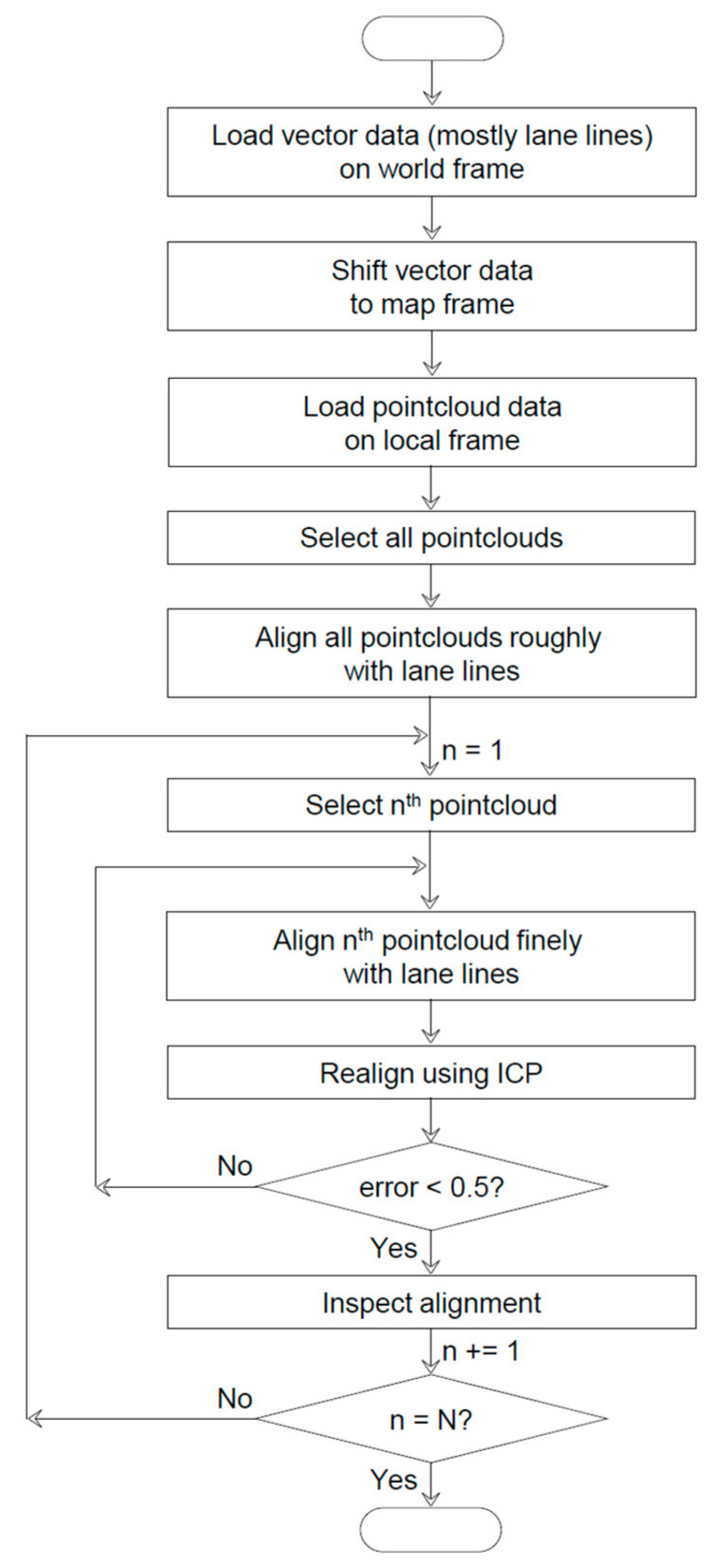
Detailed flowchart of the registration process.

The third stage is to realign the local pointcloud maps with a global vector map (see Figure 7). The realignment is performed to address the problems associated with the generated pointcloud map, which are neither sufficiently accurate nor relative to a global frame. The inaccuracy of the pointcloud map manifests as the loose ends between the first and last pointclouds, which ideally must coincide because the laser scanning ended near the point at which it started. The point at which the laser scanning started is the origin of the local pointcloud map, which must be transformed into, for example, the origin of the UTM52N zone. In the remapping process, the road surface marks in the pointcloud map are aligned with those in the vector map. The small multiple pointcloud maps produced by the approximate_ndt_mapping node facilitate stitching them together piece-by-piece on the basis of the vector map. This remapping process cannot be implemented using a large single pointcloud map.

From the database of NGII, download the vector map of an area of interest [11]. Although the coverage of HD maps has been rapidly expanded in Korea, the operational area considered in this study has not been covered. Therefore, the vector map for the Pyeong-dong industrial complex was custom-made using a dedicated mobile mapping system (MMS), while complying with the NGII vector map format. The NGII vector map is originally in the SHP format, designed to store geographic information in the form of vector data. Thus, the file format must be converted to a format readable by the software for use in the remapping process.

Launch Global Mapper to convert the SHP files representing the road surface marks to TXT files. Global Mapper is a geographic information system software that provides spatial data processing tools that can be used with a range of data formats. Under the File menu, click Batch Convert/Reproject. In the pop-up windows that are sequentially generated, select Shapefile as the filetype to perform conversion from and Simple ASCII Text as the filetype to perform conversion to. Next, add SHP files in the Source Files panel. The default options in the Destination Files panel do not need be modified, except for checking off the Include Elevations for Each Vertex box. According to the latest version of the NGII vector map format, the applicable SHP files are B2_Surfacelinemark and B3_Surfacemark, different than the applicable SHP files in the previous version (A1_LANE, A2_STOP, and B2_SURFSIGN_LINE). Click the OK button to start file format conversion. As mentioned, the road surface marks are used as references for realignment. Multi-directional references aid in realignment. For instance, lane lines are used along the *x*-axis, whereas stop lines, which are perpendicular to lane lines, are used along the *y*-axis.

Launch CloudCompare (CC) to realign the local pointcloud maps with a global vector map. CC is a 3D pointcloud editing and processing software that is especially useful for performing complex transformations on multiple pointclouds. We have screen recorded the actual usage of CC for remapping [12].

Load the converted TXT files by clicking Open under the File menu. When loading entities with coordinates greater than 10^5^ m, CC typically recommends shifting them to smaller coordinates because, by default, CC is designed to work with 32-bit values (single-precision floating-point format). The use of 32-bit values instead of 64-bit values (double-precision floating-point format) can help enhance the execution speed and decrease the memory footprint (theoretically, by 50%). In return, the 32-bit representation is subject to a limited precision. With a given number of bits, the larger the number is, the less decimals can be stored. Notably, the 32-bit representation is sufficient for entities within a local frame such as pointclouds relative to LiDAR coordinates. For example, for a laser-scanning range of 100 m, the data representation can be as precise as 10^−^^6^ m. However, if the pointclouds are relative to georeferenced coordinates, their point coordinates may be greater than 10^5^, and the data representation precision may decrease to 10^−^^2^ m. To avoid the decreased precision when loading entities with large coordinates, CC automatically suggests the best shift vector. The user may also input their own shift values that can render the first point coordinates zero. In doing so, the origin of a global frame shifts from the equator to the vicinity of the operational area, which is regarded as a map frame. As shown in [12], the coordinates of the first entity relative to the origin of the UTM52N zone are 297,127 and 3,888,656 m, and they shift to 0 and 0 m, corresponding to the origin of the map frame. Although not necessary, it is recommended to perform shifting along the *z*-axis as well, owing to its benefits in subsequent processes. Once the first shift has been made, CC uses it for the following entities.

Load the generated PCD files in the same manner. Notably, because PCD files pertain to pointclouds relative to LiDAR coordinates, their point coordinates are on the order of 100 m. Therefore, unlike TXT files, no shift is necessary. The loaded entities are stored in the DB tree on the upper left and displayed in 3D view in the middle. The global vector map and local pointcloud maps are denoted as B2_Surfacelinemark.txt and submap_*x*.pcd, respectively.

Perform rough alignment by selecting all the local pointcloud maps and aligning them with the global vector map. The loaded entities can be selected either directly in the 3D view or, preferably, through the DB tree. When clicking on a submap in the DB tree, the corresponding pointcloud map is surrounded by a bounding box in the 3D view. Multiple submaps can be simultaneously selected by pressing the Ctrl or Shift keys and simultaneously clicking on the submaps in the DB tree. Initiate the transformation mode by clicking the 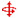 button in the upper main toolbar or Rotate/Translate under the Edit menu. After the entry, the selected entities in the DB tree are locked and the new toolstrip appears on the upper right corner of the 3D view. Four buttons are lined up in the first row of the toolstrip: (from left to right) Pause, Undo, Save and exit, and Quit without saving. The pause button is used to temporarily move out and into the transformation mode to modify the camera pose. The Undo button is used to reverse the transformation performed previously. The latter two buttons are commonly used to quit the transformation mode, and they differ only in terms of whether the previous transformations are saved. A dropdown menu in the second row is used to select the axis of rotation. Three tickboxes in the third row are used to select the direction of translation. Initiate the alignment by selecting Rotation z and check Tx and Ty in the toolstrip to translate the pointclouds in the selected submaps along the x- and y-axes and rotate them about the *z*-axis. Next, the rotations about the x- and y-axes and associated translations are performed according to a road gradient. The mouse actions used for the remapping process are presented in Table 10. Repeatedly change the pose of the pointclouds while modifying the camera pose to enhance the alignment. Perform fine alignment in the same manner as the rough registration process, albeit by selecting one single local pointcloud map and aligning it with the global vector map. Repeat this process until the last submap is aligned, previously depicted as piece-by-piece stitching.

Notably, manual alignment is cost-intensive and ineffective, owing to the large discrepancy between the two entities in terms of the number of points. For instance, a lane line is expressed by dozens of points in the pointcloud map, whereas it is expressed by only two points in the vector map. The low efficiency of manual alignment can be alleviated by implementing automatic alignment based on the iterative closest point (ICP) algorithm. Initiate the ICP by selecting a local pointcloud map and the global vector map. Subsequently, click the 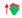 button in the upper main toolbar or Fine Registration (ICP) under the Tool menu. In the generated pop-up window, ensure that the following configuration is selected: aligned, submap_*x* and reference, B2_Surfacelinemark. This selection helps ensure that the local pointcloud map is aligned with reference to the global vector map. If not, click the swap button to reverse the maps. The default values of the parameters do not need to be altered, although the Final overlap must be decreased to 10%. As its name implies, the Final overlap specifies the actual percentage of overlap between the two pointclouds, and it functions as one of the convergence criteria. Click the OK button to execute the ICP. Although no minimum requirement for alignment accuracy exists, a root mean square error less than 0.5 can be considered acceptable. In the DB tree, .registered is appended to the name of a PCD file that has been aligned, e.g., submap_*x*.registered. The ICP is particularly helpful in performing alignment along the *z*-axis, which is considerably more challenging than alignment along the other axes. However, automatic alignment cannot always achieve superior results. The ICP may not function properly for the same reason as manual alignment. Fundamentally, the ICP is designed to iteratively minimize point-to-point distances between two pointclouds [13]. In the remapping process, the ICP selects the closest points as a correspondence and calculates the transformation matrix between the aligned (submap_*x*) and reference (B2_Surfacelinemark). However, owing to the large number of points in the pointcloud map, the corresponding points from the vector map are challenging to identify.

Consequently, the remapping process must be terminated with manual alignment as a final check. Save and export the finely aligned entity to a PCD file by clicking the 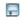 button in the upper main toolbar or Save under the File menu. Through these processes, the problems associated with the generated pointcloud map can be solved, and we obtained a pointcloud map that is sufficiently accurate and relative to a map frame.

### 2.4. Validation

The final stage is to test the registered pointcloud data using the localization module of Autoware. In doing so, prior map-based localization is realized, which is also referred to as a LiDAR localizer, in comparison with a GNSS localizer. Launch Autoware. Select the recorded pointcloud data by clicking the Ref button in the Simulation Table The ROSBAG file must be different from that used to generate the pointcloud map. Play and pause the recorded pointcloud by clicking the Play button and then the Pause button. In the Setup tab, specify Localizer by checking off the box of velodyne. Subsequently, specify Baselink to Localizer, which refers to a relative pose between the vehicle and LiDAR coordinates. The LiDAR coordinates (velodyne) are located on the back of the roof, whereas the vehicle coordinates (base_link) are located at the center of the rear axle. According to their actual location in the vehicle, specify x, y, z, yaw, pitch, and roll in meters, and then click the TF button. Before loading the registered pointcloud, downsample it using Map Tools in the Map Table Launch the pcd_filter node by pressing the PCD filter button with the default values of the parameters. Before doing this, click the Ref button and simultaneously select the produced PCD files. Downsampling decreases the size of a PCD file to 1/50 the original value. The applied Leaf Size (0.2), which can be described as a spatial sampling rate, is appended to the name of the PCD file, e.g., 0.20_submap_*x*. Load the filtered pointcloud data by clicking the Ref button and selecting the downsized PCD files simultaneously. Then, press the Point Cloud button. Next, specify TF, which indicates the relative pose between the world and map coordinates. No transformation between the coordinates is necessary because in the previous stage, the origin of the UTM52N zone has been moved to a location within the operational area. Therefore, the world frame, i.e., the global frame, is equal to the map frame. Similarly to the registered pointcloud, downsample the scanned pointcloud. In the Sensing tab, launch the downsampling node by checking off the box of voxel_grid_filter. In the pop-up window generated by clicking [app], the Voxel Leaf Size can be adjusted; however, its default value (2 m) is the most commonly used. Initiate the localization by launching the matching node by checking off the box of ndt_matching in the Computing Table In the pop-up window generated by clicking [app], the relevant parameters can be accessed. Because the default values of the parameters are predetermined for localization, they do not need to be tweaked, except for the initial pose. Unless a GNSS localizer is available, maintain the Initial Pos as zero; alternatively, specify the initial pose using the 2D Pose Estimate in RViz. Because the pose estimation is challenging using solely the scanned pointcloud, other sensors, such as cameras and GNSS receivers, can be used in the laser- scanning process to provide additional clues. Continue to publish the scanned pointcloud by clicking the Play button in the Simulation Table In RViz, confirm that the scanned pointcloud matches with the registered pointcloud. Click the RViz button. In the Displays panel, change Fixed Frame under Global Options to map, and check that the two pointclouds are overlapped.

**Figure 8:**
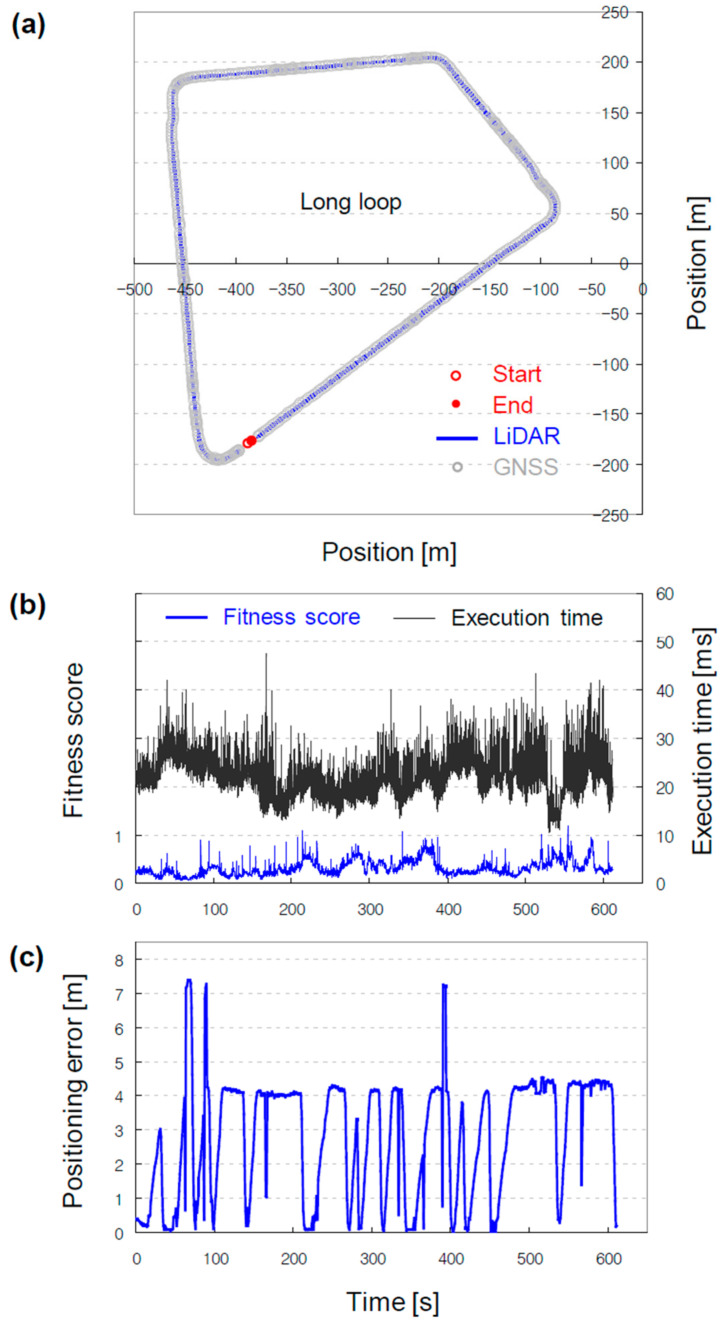
Validation results of the generated pointcloud map. (**a**) Positions of the vehicle measured by the LiDAR and GNSS localizers. (**b**) Fitness score and execution time, estimates from the LiDAR localizer, which are used as key metrics for localization based on the generated pointcloud map. (**c**) Positioning error, a measurement obtained through comparison to the GNSS localizer.

Through the localization process, the ndt_matching node publishes the current pose of the vehicle in/ndt_pose and the associated statistics in/ndt_stat, which incorporates the fitness score, execution time, and iteration number. The score indicates the degree of matching of the two pointclouds and can thus be used to infer the reliability of localization, including the quality of the generated pointcloud map. A lower score corresponds to a superior matching. GNSS receivers capable of RTK are typically used as a reference for localization; however, in this study, they were substituted by the score. The GNSS receivers were not used because they exhibit a low update rate (1 Hz), and their signals are prone to be blocked or reflected by obstacles in the operational area, such as the elevated railroad crossing the short loop. While manually driving around the long loop, the fitness score was observed to be less than one (see Figure 8b). The fine matching is indirectly evidenced by the execution time, which remained less than 40 ms. Notably, the positioning error between the LiDAR and GNSS localizers is not consistent with these statistics owing to the two reasons mentioned above. The error is primarily attributable to the low update rate. Because the low update rate caused a positioning error mostly in the longitudinal direction, the error shown in Figure 8c cannot be observed in Figure 8a.

## 3. Vector Map

**Figure 9:**
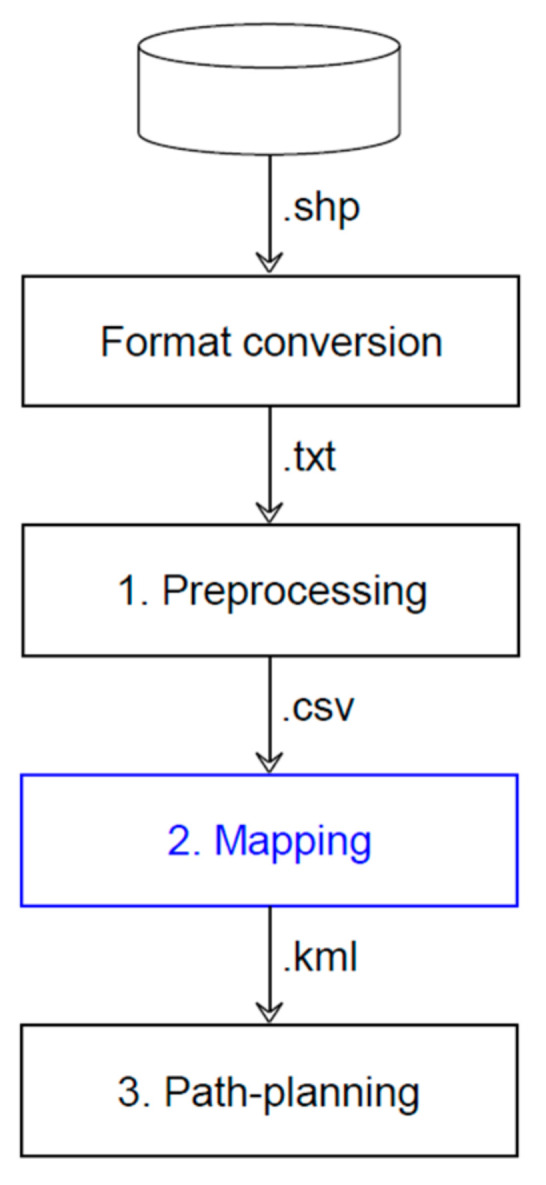
High-level flowchart outlining key stages in vector map generation.

The workflow of the vector map generation consists of three major key stages: preprocessing, mapping, and validation (see Figure 9 and Table 11).

### 3.1. Preprocessing

The first stage is to process the downloaded vector data. Among the many entities involved in vector data, lane lines are typically used for the pointcloud map. For a road network map, lane centerlines are used as a reference path. Because the NGII vector map is originally in the SHP format, it must be converted to a format readable by the software and then used in preprocessing.

As in the previous workflow, launch Global Mapper to convert the SHP file representing the lane centerline to a TXT file. Under the File menu, click Batch Convert/Reproject. In the sequentially generated pop-up windows, select Shapefile as the filetype to perform conversion from and Simple ASCII Text as the filetype to perform conversion to. Consecutively, add a SHP file in the Source Files panel. The default options in the Destination Files panel do not need to be changed, except for checking off the Include Elevations for Each Vertex and Add Blank Line Between Features boxes. According to the latest revision of the NGII vector map format, the only associated SHP file is A2_Link. Click the OK button to start the file format conversion. The converted lane centerline is not directly applicable and needs to be preprocessed to satisfy the requirements specified in OpenPlanner (see Table 12).

Notably, such preprocessing is difficult and error-prone, and in this study, a MATLAB script was written to automate the process. Run the script by typing its name, preprocess_centerlines, in the command line. The converted TXT file is input, and the script outputs as many CSV files as the number of segments. A segment denotes a set of points constituting a lane centerline. From the database of NGII, the SHP file representing the lane centerline can be downloaded by area, and thus a number of segments are mixed in random order. The points within a segment are ordered, whereas the points within a file are completely disordered. To resolve this problem, each segment is saved as a file. Based on the one file per segment, the points within a file can be arranged in sequential order. In addition, all the points on the lane centerline are regularly spaced (1 m) to serve as a waypoint. The regularly spaced points are placed wherever a global path can be generated, for instance, at the intersection. Furthermore, the points on the path are represented with the 3D position, orientation, and target speed. The points are relative to the map frame set in the previous stage to generate the pointcloud map.

### 3.2. Mapping

**Figure 10:**
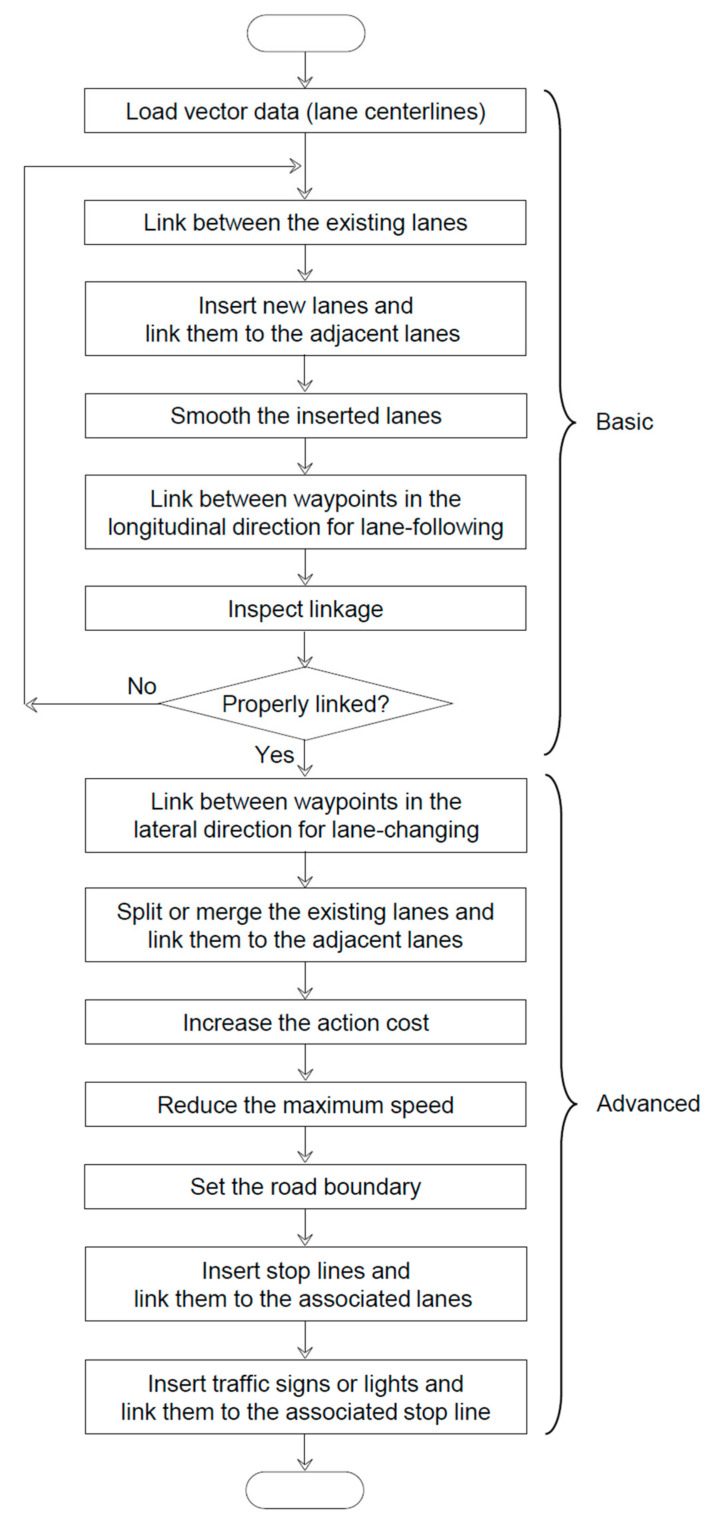
Detailed flowchart of the mapping process.

The second stage is editing of the vector data based on the processed lane centerline (see Figure 10). Because a global path and local trajectories are generated based on a lane centerline, it is considered to be the most important in path planning. However, the lane centerline must be supplemented by other vector data to render the road network map more comprehensive. Diverse entities are provided by ASSURE, including lane lines, stop lines, curbs, boundaries, crosswalks, traffic signs, and lights. As mentioned, ASSURE is an editor for a road network map, which is optimal for OpenPlanner. The lane centerline, simply called a lane in ASSURE, is the basic road network entity. The lane consists of waypoints. A waypoint contains the intrinsic information of the pose and target speed and extrinsic information such as the next, previous, left, and right waypoints. Consequently, a waypoint can be used to link different entities. The lanes can be branched or merged only through the waypoints at both ends of the lane. Details regarding the provided entities can be found in [14].

Depending on the input file formats, ASSURE can support various mapping processes (see Table 13). The mapping process selected in this study takes CSV files as inputs and outputs a KML file to be used in OpenPlanner. Additionally, PCD files are often inputted to help accurately position the entities.

Launch the ASSURE map editor. We have screen recorded the actual usage of ASSURE for the mapping [15]. The wide left panel displays all the entities in the vector map (map view), whereas the narrow right panel shows detailed information regarding the hovered-over or selected entities (info view).

#### 3.2.1. Load Lanes

Load the converted CSV files. Right-click the map view. In the generated pop-up window, hover the pointer over the Load Map and click the CSV Folder in the hover box that appears. Select the folder containing the converted CSV files and click the Open button. Confirm that the lanes are displayed on the map view.

#### 3.2.2. Link Lanes (One-to-One)

As mentioned, the loaded entities are split by file, with each file corresponding to one lane. The mapping process is initiated by linking the existing lanes on the road, described as a one-to-one link.

Click the lane; the properties of the selected lane appear under the Lane Info on the info view. Click From Lanes and press the plus sign key. In the generated blank > ID, enter the previous lane ID. In the same manner, enter the next lane ID into the blank > ID under To Lanes. After editing, press the Ctrl and S keys simultaneously. Lane Info involves many other properties other than these two properties; however, these properties do not need to be edited because they are either unused or subject to be overridden by the subsequent editing of the waypoint properties.

#### 3.2.3. Insert Lanes

Next, insert new lanes. This insertion is not necessary on the road but required at the intersection in which the lanes are not provided as an entity in the converted CSV files.

Right-click the map view. In the generated pop-up window, hover the pointer over Insert and click the Lane in the hover box that appears. Click a location at which a waypoint is desired. Add waypoints sequentially and press the enter key to insert the formed lane or the Esc key to dismiss it. Click the added waypoint and enter its z coordinate into Z, the height of which can be estimated with reference to the height of the endpoint of the lane to be linked to. To reform the lane, the existing waypoints within the lane can be moved or removed. Alternatively, new waypoints can be added. To move waypoints, click and drag the waypoint while pressing the Shift key, or click and enter the new coordinates into X and Y. To add or remove waypoints, press the plus or minus sign keys, respectively. Similar to the previous process, link the new lane to the existing lanes.

#### 3.2.4. Smooth Lanes

The inserted lanes tend to be rough because they are manually generated. Smooth the inserted lane by clicking on it and then pressing the asterisk key. Smoothing can be gradually performed by repeatedly pressing the key. The smoothing adjusts not only the position of waypoints but also the spacing between them.

#### 3.2.5. Link Lanes (One-to-Many)

Unlike the road, intersections usually require a one-to-many link. Similar to the generation of the one-to-one link, click the approaching lane; the properties of the selected lane appear under the Lane Info on the info view. Click To Lanes and press the plus sign key as many times as the number of lanes to be branched off. In the generated blanks > ID, enter the next lane IDs. After editing, press the Ctrl and S keys simultaneously.

#### 3.2.6. Link Waypoints (Longitudinal)

A linkage similar to that between lanes is also required between waypoints. Link the last waypoint on the previous lane to the first waypoint on the next lane, corresponding to a first-to-last link. Other neighboring waypoints in the middle of the lane are linked as-is.

Click the last waypoint of the previous lane; the properties of the selected waypoint appear under the Waypoint Info in the info view. From Points is already filled in. Click To Points, and press the plus sign key. In the generated blank > ID, enter the first waypoint ID of the next lane.

The opposite way around, click the first waypoint of the next lane. To Points is already filled in. In the same manner, click From Points, and press the plus sign key. In the generated blank > ID, enter the last waypoint ID of the previous lane. After editing, press the Ctrl and S keys simultaneously.

The above-mentioned processes correspond to basic mapping to solely enable lane- following. The subsequent processes describe the mapping process to allow more advanced operations including but not limited to lane-changing and traversing intersections. Notably, these operations are specific to the operational design domain corresponding to the operational area in which the AD system is designed to operate.

#### 3.2.7. Inspect Linkage

Before proceeding further, it is advisable to examine the linkages between all the drivable lanes and their waypoints. Any missing or incorrect links on the road network can degrade the global path generation ability of the global planner. In general, a larger road network is more vulnerable to error. To examine the linkages, another editor named Java OpenStreetMap (JOSM) can be used. Save and export the linked entities to an OSM file that can be read by JOSM.

Right-click the map view. In the generated pop-up window, hover the pointer over Save Map and click Lanelet2.osm in the hover box that appears. Type an OSM filename and click the Save button.

Launch JOSM. Load the linked entities by clicking Open under the File menu. In the generated pop-up window, select the converted OSM file and click the Open button. In the JOSM, the linked entities are represented as a network graph; specifically, the waypoints are displayed as nodes, and the linkage between them is displayed as an edge, allowing any missing or incorrect link to be easily found. Confirm that the waypoints are arranged sequentially.

#### 3.2.8. Link Waypoints (Lateral)

At this point, the entities are connected in the longitudinal direction. However, waypoints must also be connected in the lateral direction, enabling the global planner to facilitate lane changing.

Enter the closest waypoint ID on the left lane into the Left Point, while entering the closest waypoint ID on the right lane into the Right Point. The closest waypoint functions as a reference point when changing lanes. Therefore, it is preferable to specify a waypoint slightly ahead in the forward direction. Leave the Left and Right Points as zero for the lanes in which lane changing is not permitted by the Road Traffic Act.

#### 3.2.9. Split/Merge Lanes

After checking all the connections, split the lane into two or, oppositely, merge the two lanes, if necessary. Editing the linked lanes is useful for the behavior planner to control discrete behaviors based on the lane. The simplest example of this control is turning on a left-turn signal on a left-turn lane. To turn on the signal in advance, the lane can be slightly extended by merging the previous lane. To this end, the previous lane must not be extremely long; therefore, it must be split before being merged.

Select the waypoint that can serve as the endpoint of the separate lane. Right-click this waypoint. In the generated pop-up window, hover the pointer over Lane Functions and click the Split Line button in the hover box that appears.

The other way around, click the lane to merge the next lane. Right-click the lane. In the generated pop-up window, hover the pointer over Lane Functions and click the Merge Next Lane button in the hover box that appears. Linkage between the lanes must be ensured to merge them, and the linkage between waypoints is also crucial.

#### 3.2.10. Increase Action Cost

If necessary, increase the action cost, the default value of which is set to zero. Editing the traversal cost can help the global planner generate the optimal path. With a cost of zero, only the global path with the shortest distance is generated. However, in reality, the shortest path is not always the optimal path, for example, if it includes back streets full of illegal parking.

To optimize the path, change the action cost by clicking the lanes to be avoided. Enter the increased cost into Action Cost. The increased cost is then reflected to all the waypoints on the lane. The increased cost can be also reflected to a specific waypoint by clicking the waypoint, instead of the lane.

#### 3.2.11. Decrease Maximum Speed

If necessary, decrease the maximum speed. Editing the maximum speed can help the global planner generate a safe speed profile. Although not mentioned in the previous stage, the target speed assigned to each waypoint is determined by the maximum speed specified by the Road Traffic Act. The default value of the maximum speed is based on this value. Although the global planner uses the target speed as is, the local planner changes the speed to evade obstacles within the lane. The local planner also decreases the target speed during a turn; however, the target speed decreases as a function of the lane curvature in a tight corner. In this scenario, a vehicle may not decelerate in a timely manner during a turn. To avoid such problems, it is beneficial for the global planner to decrease the target speed in the mapping process.

Change the maximum speed by clicking the lane in which the vehicle must slow down. Enter the reduced speed as the Max Speed. The reduced speed is then reflected to all the waypoints on the lane and can be reflected to a specific waypoint by clicking the waypoint instead of the lane.

#### 3.2.12. Set Road Boundary

The road boundary must ideally be set such that the local planner only needs to react to obstacles within the lane. This setting can help address the perceptual inaccuracy. A representative example is swerving to avoid colliding with bollards. Because bollards are placed in the middle or at the end of the road, they are likely to be misidentified as obstacles within the lane. The boundary, also referred to as a wayarea, is in the form of a polygon with a set of points. To position the points accurately, the generated pointcloud map can be imported.

Right-click the map view. In the generated pop-up window, hover the pointer over the Load PointCloud button and click the Folder of .pcd(s) in the hover box that appears. Select the folder containing the relevant PCD files and click the Open button. Confirm that the registered pointcloud map is overlaid with the road network map under construction.

Again, right-click the map view. In the generated pop-up window, hover the pointer over Insert and click the Boundary button in the hover box that appears. Click the location at which a point is desired. Add points sequentially and press the enter key to insert the enclosed boundary or the Esc key to delete it. Click the added point and enter its z coordinate into Z. Although not mandatory, the height of the added point must preferably be similar to those of nearby entities. To reshape the boundary, the existing points on the boundary can be moved or removed. Alternatively, new points can be added. To move points, click and drag the point while pressing the Shift key, or click and enter its new coordinates as X and Y. To add or remove points, press the plus or minus sign keys, respectively.

#### 3.2.13. Insert Stop Lines

If necessary, insert a stop line, similar to inserting a new lane. Right-click the map view. In the generated pop-up window, hover the pointer over the Insert and click the Stop Line in the hover box that appears. The stop line can only consist of two endpoints. Click the location at which one endpoint is desired. By adding the other endpoint, the stop line can be approximately located.

To accurately locate the stop line, revert to the first stage. Launch Global Mapper to convert the SHP file representing the stop line to a CSV file. The applicable SHP file is B2_Surfacelinemark or A2_STOP, depending on the version of the NGII vector map format.

Load the converted CSV file and save it as a KML file. Right-click the map view. In the generated pop-up window, hover the pointer over the Save Map button and click ASSURE.kml in the hover box that appears. Type a KML filename and click the Save button. Continue to run the ASSURE map editor to load the converted KML file and identify the position of the stop line. Right-click the map view. In the generated pop-up window, hover the pointer over Load Map, and click the Merge Items .kml button in the hover box that appears. Note that the loaded stop line is not a part of the road network map being constructed and used only to evaluate its position.

Click the endpoint; the properties of the selected endpoint appear under the Point Info in the info view. Using the X, Y, and Z of the endpoint, the approximately located stop line can be relocated. Ensure that the finely located stop line sufficiently reaches the lane to be connected to. If not, extend the lane. Click the stop line; the properties of the selected stop line appear under the Stop Line Info in the info view. The stop line and lane are linked by entering the lane ID and stop line ID into the Lane ID and SL ID, respectively. To activate the linked stop line, it must be joined again to either the traffic sign or lights, depending on the presence of traffic lights at the intersection.

#### 3.2.14. Insert Traffic Signs

If the stop line is located at a non-signalized intersection, insert the traffic sign. Right-click the map view. In the generated pop-up window, hover the pointer over Insert, and click the Sign button in the hover box that appears. The Sign button is used to specify the position and type of the traffic sign. Only the stop sign is available at the present. Click to set the position of the traffic sign and click again to set its orientation. Click the traffic sign; the properties of the selected traffic sign appear under Sign Info in the info view. The traffic sign ID is assigned to Sign ID. Click Type and press the right arrow key to select the stop sign. Click the associated stop line and enter the Sign ID into Stop Sign ID. To relocate the stop sign, click on it. The sign is represented by a rectangle with a midline and two points. The large point is within the rectangle, and the small point is in the orbit around the large point. To move the stop sign, click and drag the large point while pressing the Shift key, or click and enter its new coordinates into X, Y, and Z. To rotate the stop sign, click and drag the small point while pressing the Shift key.

#### 3.2.15. Insert Traffic Lights

If the stop line is located at a signalized intersection, insert the traffic light. Right-click the map view. In the generated pop-up window, hover the pointer over Insert and click the Light button in the hover box that appears. The Light button is used to specify the position and type of the traffic light bulb. The signal types for vehicles are green, yellow, red, left, and right and those for pedestrians are green and red. Similar to the traffic sign, click to set the position of the traffic light bulb and click it again to set its orientation. The positions of traffic lights must be as close to the real situation as possible because the TLR in the developed AD system requires the accurate location of the traffic lights on the road network map. Click the traffic light bulb; the properties of the selected traffic light bulb appear under Traffic Light Info in the info view. The traffic light ID is assigned to TL ID. Click Type and press the right arrow key to select the relevant signal type. Enter the associated stop line ID into Stop Line. In addition, click Controlled Lanes, and press the plus sign key. In the generated blanks > LID, enter the associated lane IDs. The opposite way around, click the associated stop line. Click Related Lights, and press the plus sign key as many times as the number of the traffic light bulbs to be linked. In the generated blanks > LID, enter the associated traffic light IDs. The traffic light can be relocated in the same fashion as the traffic sign.

### 3.3. Validation

The final stage is to test the edited vector data using the path-planning module of Autoware. As mentioned, OpenPlanner is a representative path-planning module within Autoware [16]. The module incorporates global, local, and behavior planners that are implemented as a series of ROS nodes (see Table 14). The global planner uses the traversal cost to determine the optimal path. Based on the global path, the local planner uses the collision cost to determine the best trajectory among candidate trajectories, also referred to as roll-outs. To this end, the lidar_kf_contour_tracker node from the detection module and op_motion_predictor node are engaged between the op_trajectory_generator node and op_trajectory_evaluator node in the path-planning module.

The global planner uses the traversal cost to determine the optimal path. Based on the global path, the local planner uses the collision cost to determine the best trajectory among the candidate trajectories, also referred to as the roll-outs. To this end, the lidar_kf_contour_tracker node from the detection module and op_motion_predictor node are engaged between the op_trajectory_generator node and op_trajectory_evaluator node in the path-planning module.

Launch Autoware. The actual usage of Autoware for path planning has been screen recorded in [17]. As in the previous validation process, specify Localizer and Baselink to Localizer in the Setup Table The Vehicle Model and Vehicle Info can be optionally provided. Instead of loading the edited vector data by pressing the Vector Map button in the Map tab, edit the launch file of the op_global_planner node. Set the mapSource argument to two (KML file) and provide its path and filename to the mapFileName argument. Revert to the Map tab, and specify TF. In the Computing tab, launch the connector node by checking off the box of vel_pose_connect. In the pop-up window generated by clicking [app], check off the Simulation Mode box and press the OK button. As discussed, the current pose is measured by the matching node. The current velocity is measured from the vehicle side and transmitted to the connector node via the CAN bus. In simulation mode, these measurements are replaced with the estimates provided by the simulator built in Autoware, which is known as the wf_simulator. Launch the tracker node by checking off the kf_contour_tracker box. Launch the global planner node by checking off the op_global_planner box. In the pop-up window that is generated by clicking [app], check off the RViz Goals box and press the OK button. In doing so, the user can specify the start and goal poses in RViz. In addition, check off the Enable Smoothing, Enable Replanning, and Enable Lane Changing boxes. Click the RViz button. Confirm that the generated road network map is displayed. In the Displays panel, check off the Vector Map Centerlines box to display the lane edited in the previous stage. The name may differ according to a configuration file (/vector_map_center_lines_rviz is published). Press the 2D Pose Estimate and Nav Goal buttons to set the start and goal poses, respectively. The goal pose can be set multiple times to allow the vehicle to navigate consecutively with replanning. By doing this, the vehicle starts for the next goal as soon as it reaches the current goal. If the Global Path box in the Displays panel is checked, the generated global path is displayed.

**Figure 11:**
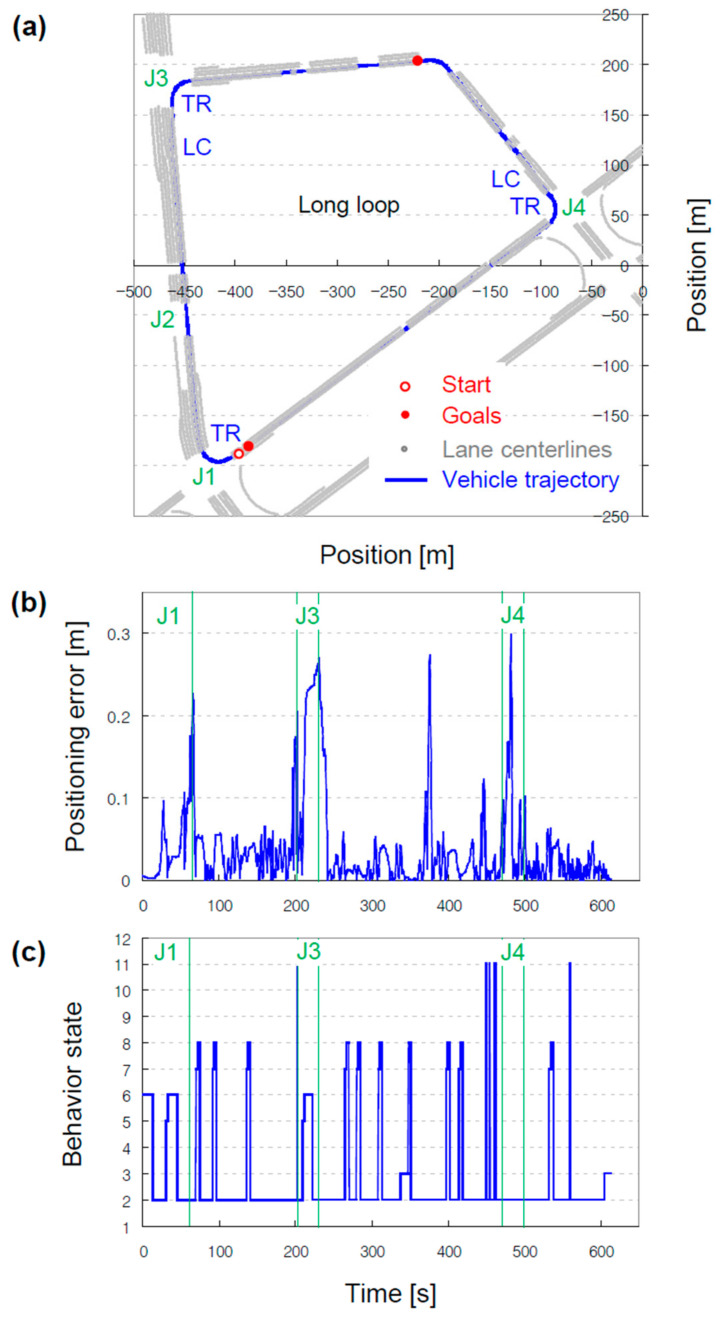
Validation results of the generated road network map. (**a**) Vehicle trajectory from the start to goal locations, generated based on lane centerlines. J, LC, and TR refer to junction, lane changing, and right turn, respectively. (**b**) Positioning error, a measurement by comparing the current positions of the vehicle and its nearest waypoint. (**c**) Behavior state transition according to operations supported by the generated vector map.

Repeatedly send the op_global_planner node all possible start and goal locations, and see if the optimal path can be generated as a result of global planning based on the created vector map. As previously mentioned, the operational area consists of main streets without back streets. Hence, the global planning requires only a simple validation, and the shortest path is the optimal path. In the operational area, the long loop is preferable to the short loop because in the long loop, considerable validation is required for local and behavioral planning, such as lane changing before traversing the signalized intersection. Starting from a one-way road between the long and short loops, the global path around the long loop can be generated by setting an intermediate goal; otherwise, the global path is generated pertaining to the short loop (see Figure 11a).

Launch the local planner nodes by checking off op_common_params, op_trajectory_generator, op_motion_predictor, and op_trajectory_evaluator. In the pop-up window that is generated by clicking [app], check off the Enable Prediction box, and press the OK button. In doing so, the op_trajectory_evaluator node can use the estimated trajectories of dynamic obstacles and their measured contour to calculate the collision cost. The calculated cost is reflected to the generated trajectories of the vehicle, which can be represented by color. Generally, a darker red corresponds to a higher cost. Therefore, the red trajectory represents the fully blocked trajectory with the highest cost, whereas the pink trajectory indicates the optimal trajectory with the lowest cost. As the final node in the local planning pipeline, launch the behavior planner node by checking off the box of op_behavior_selector. Launch the filter node by checking off the twist_filter box, and launch the waypoint follower node by checking off the pure_pursuit box. The pure_pursuit node publishes the twist command to the twist filter node in/twist_raw. The twist filter node consecutively publishes the modified command in/twist_cmd by smoothening and limiting the linear and angular velocities. Provide the velocity command, and check if the vehicle can follow the best trajectory generated as a result of local planning based on the created vector map. Additionally, see if the vehicle can handle discrete behaviors, such as avoiding dynamic obstacles and stopping before a stop line or traffic lights in consequence of behavior planning.

The main test scenarios to validate the generated vector map were possible only with the wf_simulator. This is because lane following, lane changing, and intersection traversal can be performed without dynamic obstacles. Although not the main test scenario, swerving to avoid dynamic obstacles was impossible due to the limited functionality of the wf_simulator. For more rigorous and comprehensive validation, state-of-the-art simulators, such as CARLA [18], LGSVL [19], and CarMaker [20,21,22,23] are required. We instead performed physical tests in the operational area.

While autonomously driving around the long loop, the positioning error between the reference and actual trajectories was observed to be less than 0.1 m, except near intersections (see Figure 11b). As mentioned, the reference trajectory was generated based on lane centerlines in the created vector map, and the actual trajectory followed by the vehicle was measured by the LiDAR localizer. The error can be attributed to the difference in the current positions of the vehicle and its nearest waypoint. The long loop had four signalized intersections (J1 to J4), and the vehicle turned right at J1, J3, and J4. To avoid illegal parking, the vehicle drove in the middle lane and moved to the far-right lane when approaching the intersection. The error increased during lane changing because the vehicle moved between waypoints on the lanes. Moreover, the error increased when turning right because the vehicle was steered by more than the amount referenced by the local planner. This behavior depended on the look-ahead distance in the pure_pursuit node. The behavior states transitioned from/to FORWARD (2), STOPPING (3), TRAFFIC_LIGHT_STOP (5), TRAFFIC_LIGHT_WAIT (6), STOP_SIGN_STOP (7), STOP_SIGN_WAIT (8), FOLLOW (9), and OBSTACLE_AVOIDANCE (11) (see Figure 11c). Among the four signalized intersections in the long loop, the vehicle stopped at a red light at J1 and J3. Moreover, the vehicle stopped at both the vehicle and pedestrian traffic lights at J1, whereas it stopped only at the pedestrian traffic lights at J3. In the case of the signalized intersections, the state proceeded with 2, 3, 5, 6, and 2. In the case of the non-signalized intersections, states 5 and 6 were replaced by states 7 and 8, respectively. The vehicle almost stopped and swerved to avoid colliding with a long truck parked near J4, and the state proceeded accordingly with 2, 9, 11, and 2.

## 4. Conclusions

In this paper, we presented two separate but successive HD map generation workflows for pointcloud and vector maps. The generated HD maps were implemented into the localization and path-planning modules, respectively. The implemented HD maps were validated in the AD system of an environmental-monitoring vehicle. Exploiting the HD maps, we obtained a provisional driving permit by passing a screening test held at K-City, an unpopulated city for autonomous vehicles. K-City is equipped with five major test environments: high-speed, urban, rural, pedestrian-centric roads, and a parking lot. The driving permit was required to deploy the vehicle to the operational area, the Pyeong-dong industrial complex in Gwangju-si. The Ministry of Land, Infrastructure, and Transport in Korea has revised the provisional driving permit regulations. To facilitate the development of unmanned vehicles, their types are subdivided into A, B, and C. Type C does not require attendants to operate. The environmental-monitoring vehicle is of type C, and it was recorded as the first vehicle to obtain the type C driving permit, according to the revised regulations. Using the HD maps, we could subsequently achieve the accumulated mileage of 200 km with no intervention in the operational area.

Future work is needed to generalize the HD map format. Although we selected ASSURE KML, owing to its compatibility with the path-planning module, more standardized PSFs such as OpenDRIVE will be required to render the AD system more expandable. As described, the HD maps were generated based on entities involved in the NGII vector map: B2_Surfacelinemark and B3_Surfacemark for the pointcloud map and A2_Link for the vector map. Therefore, map format conversion will be necessary, for instance, between NGII and OpenDRIVE.

## Figures and Tables

**Table 1:** Evolution of maps for navigation (adapted from [1]).

Year	Type	Dimension	Level	Accuracy
1930	Paper maps	2D	Road	
1990	Digital maps	2D	Road	5–10 m
2000	Enhanced digital maps	2D/3D	Road/Lane	50 cm
2010	High-definition maps	3D	Lane	10–20 cm

**Table 2:** Comparison between digital and HD maps (adapted from [2]).

		Digital Maps	HD Maps	Notes
Survey method		Aerial imagery	Mobile mapping system	
Dimensions		2D	3D	
Accuracy	Horizontal	3.5 m (0.7 m)	0.25 m	1:5k (1:1k) scale
	Vertical	1.67 m (0.33 m)	0.25 m	
Entities	Lane line	X	O	
	Lane centerline	X	O	
	Lane boundary	O	O	
	Surface mark	X	O	
	Traffic sign	△	O	Urban area, location only
	Traffic lights	X	O	

**Table 3:** List of layers comprising an LDM (adapted from [4]).

Layer	Time Interval	Contents
Highly dynamic	Less than several seconds	Positions and states of pedestrians, vehicles, motorbikes, etc.
Transient dynamic	Less than several minutes	Positions and states of obstacles (illegally parked vehicles), local weather (unexpected heavy rain), etc.
Transient static	Less than several hours	Positions and states of road works, lane closures, broken-down vehicles, accident sites, etc.
Permanent static	A day or longer	Positions of lanes, traffic signs and lights, etc.

**Table 4:** Comparison between Aisan vector map and major PSFs for use in Autoware (adapted from [5]).

PSF	Advantages	Limitations
Aisan Vector Map	Successfully adopted by Autoware ROS package to read map data	Proprietary map format Not widely adopted
OpenDRIVE	Standardized by the ASAM system	Few tools to write map data
Lanelet2 with OSM XML	Easy to create using nodes as geometric primitives	Many OSM tools but few tools for Lanelet2
NDS Open Lane Model	Standardized under the NDS association Directly used in production	Prohibitively expensive license

**Table 5:** Specification of instruments used for environmental monitoring.

Analysis Target		Model	Manufacturer	Method
Air	SO_2_	T100	Teledyne API	UV fluorescence
	NO/NO_2_/NO_X_	T200		Chemiluminescence
	CO	T300		Gas filter correlation
	O_3_	T360M		Mid-range gas filter correlation
	CO_2_	T400		UV absorption
	Dust	11-D	GRIMM	Aerosol spectroscopy
Road surface		In-house developed	UOK	Deep learning-enabled computer vision

**Table 6:** Latest revision of NGII vector map format (adapted from [7]).

Entity Code_Name	Type	Description
A1_Node	Point	Points connecting lane centerline
A2_Link	Line	Lane centerline
A3_Drivewaysection	Polygon	Road facilities (tunnel, bridge, overpass, underpass, etc.)
A4_Subsidlarysection	Polygon	Roadside facilities (rest area)
A5_Parkinglot	Polygon	Parking lot as roadside facilities
B1_Safetysign	Point	Traffic signs (stop and give way, no entry, no parking, road work, roundabout, speed limit, wild animals, etc.)
B2_Surfacelinemark	Line	Line-shaped road surface marks (lane line, stop line, etc.)
B3_Surfacemark	Polygon	Polygon-shaped road surface marks (crosswalk, etc.)
C1_Trafficlight	Point	Traffic lights
C2_Kilopost	Point	Traffic sign indicating the distance from the starting point of the road
C3_Vehicleprotectionsafety	Line	Road facilities preventing vehicles from driving off the road
C4_Speedbump	Polygon	Speed bump
C5_Heightbarrier	Line	Road facilities deterring high vehicles from entering the underpass.
C6_Postpoint	Point	Poles of traffic signs and lights

**Table 7:** Four stages of pointcloud map generation.

Stage	Toolset	Input File Format	Output
Laser scanning	Autoware (LiDAR driver node)		Recorded pointcloud
Mapping	Autoware (approximate_ ndt_mapping node)	ROSBAG	Aligned pointcloud on a local frame
Registration	Global Mapper CloudCompare	SHP, TXT PCD	Aligned pointcloud on a global frame
Validation	Autoware (ndt_matching node)	ROSBAG PCD	Pointcloud map applicable to localization

**Table 8:** Description of the operational area with the number of lanes and road facilities.

Loop	Road	Length	Lanes	Stop Lines	Traffic Lights	Crosswalks
Long	L1	399.8 m	8	4	3	3
	L2	274.6 m	4	4	1	3
	L3	195.5 m	4	4	4	2
Long/Short	LS	442.7 m	2	2	2	2
Short	S1	86.8 m	1	1	0	1
	S2	355.9 m	3	0	0	0
	S3	86.8 m	1	1	0	1

**Table 9:** Comparison of mapping nodes provided in Autoware.

	ndt_mapping	approximate_ndt_mapping
Input	Scanned pointcloud	
Algorithm	Normal distributions transform	
Output	Larger single pointcloud	Smaller multiple pointclouds
Advantages	Can be more accurate	Can map a larger area Requires less computation and less memory
Limitations	Requires more computation to align the latest pointcloud with all previous pointclouds and more memory to store all the pointclouds	Might be less accurate

**Table 10:** Comparison of mouse actions in versus out of the transformation mode.

	In the Transformation Mode	Out of the Transformation Mode
Purpose	To modify the pose of pointclouds	To modify the camera pose
Left-click and hold	Rotation	Position
Right-click and hold	Translation	Orientation
Scroll up and down	Zoom in and out	

**Table 11:** Three stages of vector map generation.

Stage	Toolset	Input File Format	Output
Preprocessing	Global Mapper MATLAB	SHP TXT	Processed lane centerlines (see Table 12 for more details)
Mapping	ASSURE	CSV PCD	Linked lanes and waypoints Adjusted action cost and max speed Fixed boundary Linked stop line, traffic signs and lights
Validation	Autoware (multiple ROS nodes in OpenPlanner)	KML KML.PROJ.DAT	Vector map applicable to path-planning

**Table 12:** Requirements for using A2_Link as source data of a global path in OpenPlanner.

A2_Link	Requirements
Consists of only two points at both ends	Several equally spaced points that can serve as waypoints in a global path
Points are represented only by their position	Plus the orientation between adjacent points, target speed assigned onto each point
Points are only ordered within a segment and unordered among different segments. With multiple segments per file (one single SHP file), points are unordered within a file.	With one file per segment (multiple CSV files), points are ordered within a file.
Only available on the road	At the intersection
Covers the entire area	Can select only the area in need
Relative to the world frame (UTM52N)	The map frame

**Table 13:** List of function and input and output file formats provided in ASSURE.

Function	Input	Output File Format
Create	From scratch	KML (OpenPlanner) KML (Google Earth) OSM (Lanelet2)
	CSV	
	Guided by PCD	
Import and edit	KML (OpenPlanner)	
	OSM (Lanelet2)	
	XORD (OpenDRIVE)	
	CSV (Aisan Vector Map)	
Merge	Multiple KMLs	One single KML

**Table 14:** List of ROS nodes comprising OpenPlanner and their main inputs and outputs.

Module	ROS Node	Main Input	Main Output (ROS Topic)
Path planning (Global planner)	op_global_planner	Start pose Goal poses Vector map	Global path (/lane_waypoints_array)
Path planning (Local planner)	op_common_params		Common parameters for the local planner
	op_trajectory_generator	Global path	Local trajectories (/local_trajectories)
Detection	lidar_kf_contour_tracker	Detected objects	Tracked objects (/tracked_objects)
Path planning (Local planner)	op_motion_predictor	Tracked objects	Predicted objects (/predicted_objects)
	op_trajectory_evaluator	Local trajectories Predicted objects	Best local trajectory (/local_weighted_trajectories) Local trajectory cost (/local_trajectory_cost)
Path planning (Behavior planner)	op_behavior_selector	Best local trajectory Local trajectory cost	Behavior states (/op_current_behavior) Final waypoints (/final_waypoints)
